# Hydrogen Sulfide Prolongs Postharvest Storage of Fresh-Cut Pears (*Pyrus pyrifolia*) by Alleviation of Oxidative Damage and Inhibition of Fungal Growth

**DOI:** 10.1371/journal.pone.0085524

**Published:** 2014-01-15

**Authors:** Kang-Di Hu, Qian Wang, Lan-Ying Hu, Shuai-Ping Gao, Jun Wu, Yan-Hong Li, Ji-Lian Zheng, Yi Han, Yong-Sheng Liu, Hua Zhang

**Affiliations:** 1 School of Biotechnology and Food Engineering, Hefei University of Technology, Hefei, Anhui Province, China; 2 College of Horticulture, Nanjing Agricultural University, Nanjing, Jiangsu Province, China; Key Laboratory of Horticultural Plant Biology (MOE), China

## Abstract

Hydrogen sulfide (H_2_S) has proved to be a multifunctional signaling molecule in plants and animals. Here, we investigated the role of H_2_S in the decay of fresh-cut pears (*Pyrus pyrifolia*). H_2_S gas released by sodium hydrosulfide (NaHS) prolonged the shelf life of fresh-cut pear slices in a dose-dependent manner. Moreover, H_2_S maintained higher levels of reducing sugar and soluble protein in pear slices. H_2_S significantly reduced the accumulation of hydrogen peroxide (H_2_O_2_), superoxide radicals (•O_2_^−^) and malondialdehyde (MDA). Further investigation showed that H_2_S fumigation up-regulated the activities of antioxidant enzymes ascorbate peroxidase (APX), catalase (CAT), and guaiacol peroxidase (POD), while it down-regulated those of lipoxygenase (LOX), phenylalanine ammonia lyase (PAL) and polyphenol oxidase (PPO). Furthermore, H_2_S fumigation effectively inhibited the growth of two fungal pathogens of pear, *Aspergillus niger* and *Penicillium expansum*, suggesting that H_2_S can be developed as an effective fungicide for postharvest storage. The present study implies that H_2_S is involved in prolonging postharvest storage of pears by acting as an antioxidant and fungicide.

## Introduction

Hydrogen sulfide (H_2_S), traditionally been thought of as poisonous gas, has proved to be a gaseous signaling molecule in animals [1], [2]. Accumulating evidence unveiled its role as a gaseous regulator involved in various processes in plants, including seed germination, root organogenesis, abiotic stress tolerance, guard cell movement and autophagy [3], [4], [5], [6], [7], [8]. H_2_S can be generated from cysteine or sulfite by the enzymatic actions of O-acetylserine (thiol) lyase or sulfite reductase respectively, further suggesting that it might act as an endogenous regulator in plants [9]. In our previous studies, we found the role of H_2_S in delaying senescence of cut flowers in a wide spectrum of botanical species including herbaceous and woody plants [10]. More recently, H_2_S was found to prolong the postharvest shelf life of nonclimacteric fruit strawberry by acting as an antioxidant [11]. The concentration of the applied H_2_S required to delay senescence in strawberry is quite low and the endogenous content of H_2_S in H_2_S-fumigated strawberry is comparable to that of control fruit, suggesting that H_2_S fumigation to fruits could be safe [11]. However, whether H_2_S is implicated in ripening and senescence of climacteric fruits remains unknown.

Pear (*Pyrus pyrifolia*) is one of the most economically important fruit crops in temperate zones, but has a limited postharvest life. Pear is a typical climacteric fruit, where the respiratory climacteric precedes rapid fruit ripening. Many physiological disorders of pear fruit and microbial caused rot, appear during fruit ripening in storage and are considered to be associated with fruit senescence [12], [13]. Postharvest fruits undergo programmed senescence accompanied by various physiological and biochemical changes, including oxidative damages caused by reactive oxygen species (ROS) such as hydrogen peroxide (H_2_O_2_) and superoxide radicals (•O_2_^−^) [14]. ROS are highly reactive and cause lipid peroxidation, which could lead to undesirable flavors and odors due to increased lipoxygenase (LOX) activity and malondialdehyde (MDA) content in fruit tissue [15], [16]. Antioxidant enzymes such as ascorbate peroxidase (APX), catalase (CAT) and peroxidase (POD) are applied to degrade ROS to cope with the oxidative challenge during fruit senescence [17]. The strategies to alleviate oxidative senescence of climacteric fruits included treatments with ROS antagonists, nitric oxide (NO) or 1-methylcyclopropene (1-MCP) during postharvest storage of peach (*Prunus persica* L.) and mango (*Mangifera indica* L.) [18], [19], [20], [21]. NO could delay ripening and improve the post-cutting quality of banana (*Musa balbisiana* Colla), peaches and apple (*Malus pumila* Mill.) [22], [23], [24]. Due to the similar functional characteristics of NO and H_2_S in animal system and plants [1], [8], we speculate that H_2_S might also be involved in maturation and senescence regulation in fruit.

NO was found to inhibit the growth of pathogens on postharvest fruit and vegetables [25]. In plants, L-cysteine desulfhydrase expression and activity which are responsible for H_2_S release are induced upon pathogen attack [26], further suggesting that the released H_2_S has a role in plant defence. Besides, inorganic and organic sulfur compounds have long being used as fungicides. Considering the similar physiological roles of H_2_S and NO [1], [8], we hypothesize that H_2_S has similar inhibitory effect on the growth of microbial pathogens in the background of fruit storage. Therefore, in the present study, we characterized the physiological responses of pears to H_2_S fumigation during storage and the inhibitory role of H_2_S on the growth of fungi isolated from pears. We show that H_2_S alleviated the decay of pear slices probably by an enhanced antioxidant system to eliminate excessive ROS and by inhibiting fungal infection on pears.

## Results

### H_2_S alleviates the decay of fresh-cut pears

**Figure 1:**
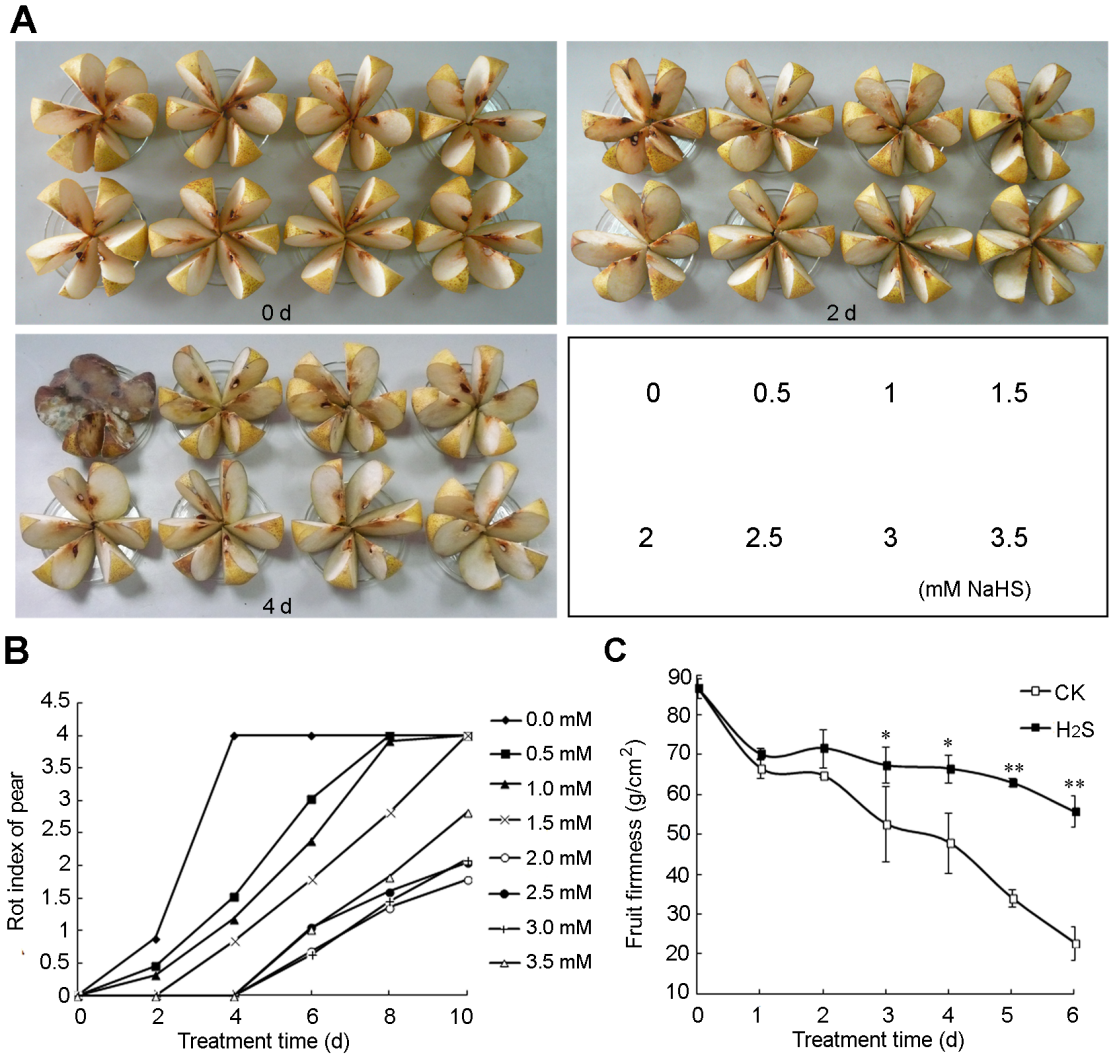
Hydrogen sulfide (H_2_S) delays the rotting of fresh-cut pear in a dose-dependent manner. Pear slices were fumigated with H_2_S released from different concentrations of NaHS as shown in the lower right part of photographs (A), and the photographs were taken on day 0, 2 and 4, and the rot index (B) of pear slices was recorded every two days from day 0 to 10. (C) Change of firmness in fresh-cut pear for water treatment (CK) and 2 mM NaHS treatment (H_2_S) during storage at 20°C for 0−6 days. Data are presented as means ± SD (n = 6). * and ** in this figure and following ones stand for significant difference between CK and H_2_S at P<0.05 and P<0.01, respectively.

H_2_S, released by H_2_S donor NaHS aqueous solutions, was applied to pear slices. As shown in Figure 1A, H_2_S prolonged the shelf life of fresh-cut pears and alleviated flesh mealiness in a dose-dependent manner. Rot index of control tissue treated with water increased significantly after 2 day of storage and reached a maximum value on day 4. In contrast, rot index of pear slices treated with H_2_S was lower than water controls even after 4 days of storage (Figure 1B). The shelf life of pear slices treated with 2.0 mM NaHS was extended to 8 d, while concentrations higher than 2.0 mM showed no further positive effect. As shown in Figure 1C, pear slices showed a decrease in firmness with storage time in both water controls and NaHS treatment. However, fruits treated with 2 mM NaHS were significantly harder than the control group during the storage period (Figure 1C).

### H_2_S increases the contents of soluble protein and reducing sugar in pear slices

**Figure 2:**
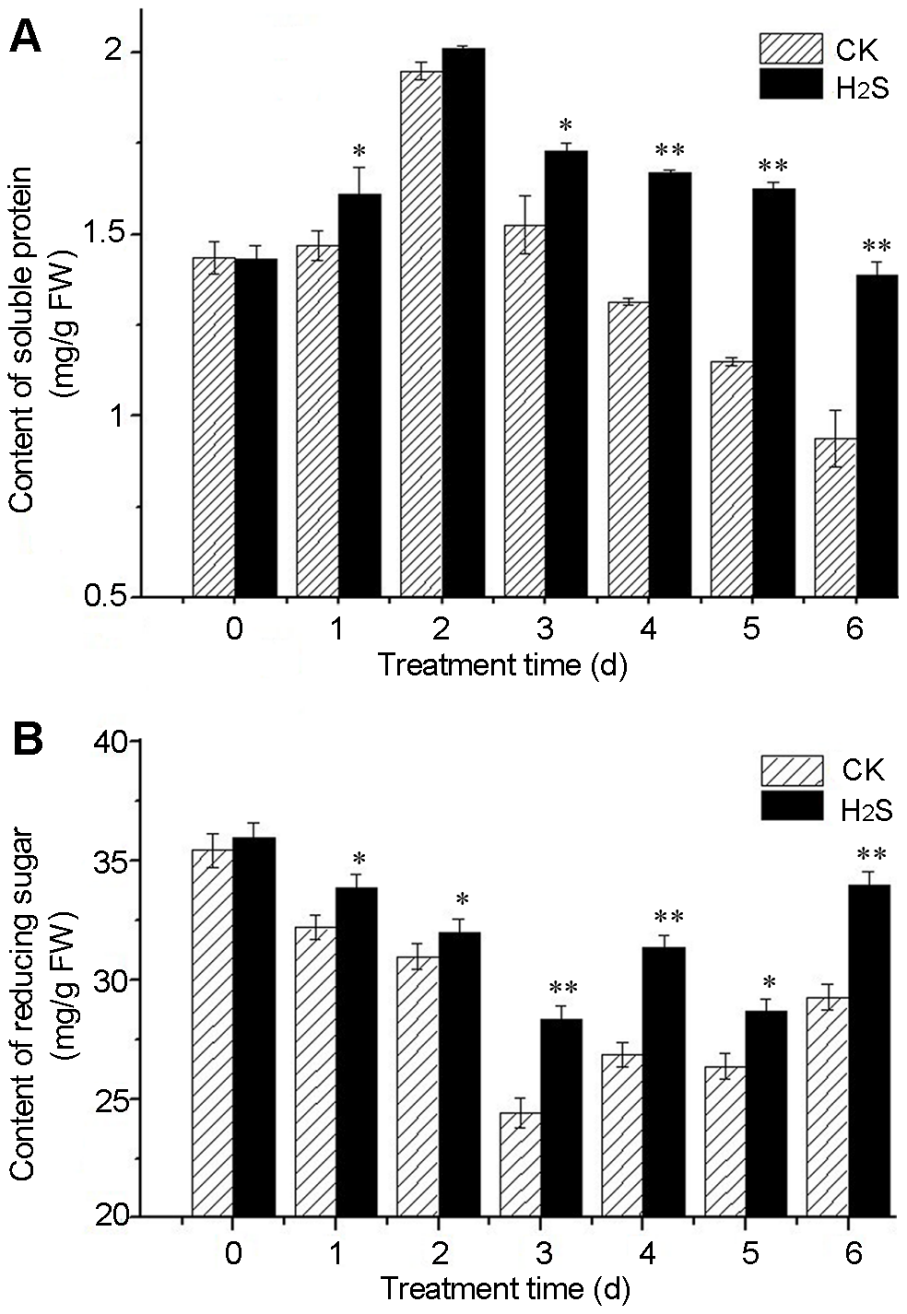
H_2_S increases (A) soluble protein and (B) reducing sugar in pears. Soluble protein and reducing sugar were measured in slices treated with H_2_O (CK) or 2.0 mM H_2_S donor, NaHS (H_2_S) for 0−6 days. FW: fresh weight.

The data in Figure 2 indicated that H_2_S treatment sustained higher level of soluble protein and reducing sugar in pear slices during storage compared with water controls. The content of soluble protein in pear slices increased during the first 2 d of storage regardless of treatments. Thereafter the content of soluble protein declined rapidly in water controls, while H_2_S treatment attenuated such decline (Figure 2A). The levels of reducing sugar in pear slices decreased in the first 3 days in both treatments, then increased gradually (Figure 2B). During the whole storage, H_2_S treatment sustained higher level of reducing sugar than water controls.

### Changes of free amino acids in pears during storage

Table 1 shows that total amino acids in pear slices on 3 d of storage increased by ∼44% in water controls relative to fresh tissue. In contrast, H_2_S treatment for 3 days maintained comparable level of total free amino acids as in fresh pear slices. There were pronounced differences in the amino acid profiles in pear slices treated with H_2_S compared with water control. Cysteine, glutamic acid, histidine and alanine were all higher in H_2_S-treated tissue than those of water control, whereas threonine, methionine, isoleucine, tyrosine, phenylalanine and arginine were not detected in pear slices regardless of treatments.

**Table 1:** Effects of H_2_S treatment on the content of free amino acids (nmol/g FW) in pear slices determined after 3 days of storage at 20°C.

	Lys	His	Ala	Leu	Val	Asp	Ser	Glu	Gly	Cys	Thr	Met	Ile	Tyr	Phe	Arg	total
0	34.2	ND	215.4	77.9	129.5	1041.1	3225.5	10.2	32.9	ND	ND	ND	ND	ND	ND	ND	4766.8
CK	37.9	65.2	235.9	69.5	97.4	1609.4	4707.1	ND	19.5	ND	ND	ND	ND	ND	ND	ND	6841.8
H_2_S	ND	66.2	489	56.7	96.9	817.9	2760.7	73.3	14	1.3	ND	ND	ND	ND	ND	ND	4414

0 stands for fresh-cut pear slices; pear slices were treated with water (CK) or 2.0 mM H_2_S donor NaHS (H_2_S) for 3 days.

### H2S down-regulates •O_2_^−^, H_2_O_2_ and MDA contents in pears

**Figure 3:**
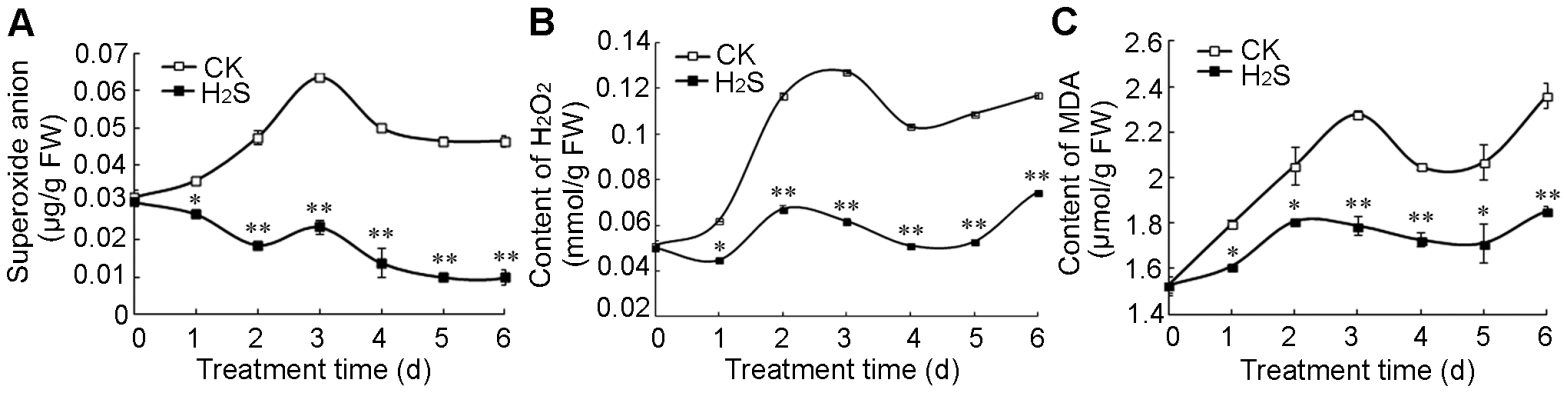
H_2_S down-regulates superoxide anion (•O_2_^−^) (A), hydrogen peroxide (H_2_O_2_) (B) and malonaldehyde (MDA) (C) in pears. Pear slices were fumigated with 2_2_S donor NaHS aqueous solution (H_2_S) or with water as the control groups (CK) for 0−6 d. Data are presented as means ± SD (n = 3). FW: fresh weight.

Figure 3 shows that H_2_S fumigation sustained significantly lower levels of •O_2_^−^ (A), H_2_O_2_ (B) and MDA (C) than water controls. The •O_2_^−^ content of pear slices in water controls increased rapidly during the first 3 d of storage, followed by a slight decrease. In contrast, •O_2_^−^ levels in H_2_S-treated pear slices decreased gradually from 0.03 µg/g in fresh tissues to 0.01 µg/g on day 5 and 6 (Figure 3A).

Similar change variations of H_2_O_2_ were observed in water controls and H_2_S treatment (Figure 3B). H_2_O_2_ in water controls accumulated quickly during the first 3 days of storage then kept a high stable level until the end. However H_2_S treatment maintained significantly lower level of H_2_O_2_. For instance, H_2_O_2_ content in water controls was about two-fold that of pear slices treated with H_2_S on day 3, 4 and 5 days (Figure 3B).

H_2_S treatment also kept a significantly lower level of MDA compared with water controls (Figure 3C). MDA content in water controls increased rapidly during the first 3 d of storage, while in H_2_S-treated pear slices, MDA accumulated much more slowly. Thereafter, MDA content of pear slices showed little variation with time in the presence or absence of H_2_S (Figure 3C).

### H2S up-regulates the activities of APX, CAT, POD, and down-regulates the activities of LOX, PAL and PPO in pears

**Figure 4:**
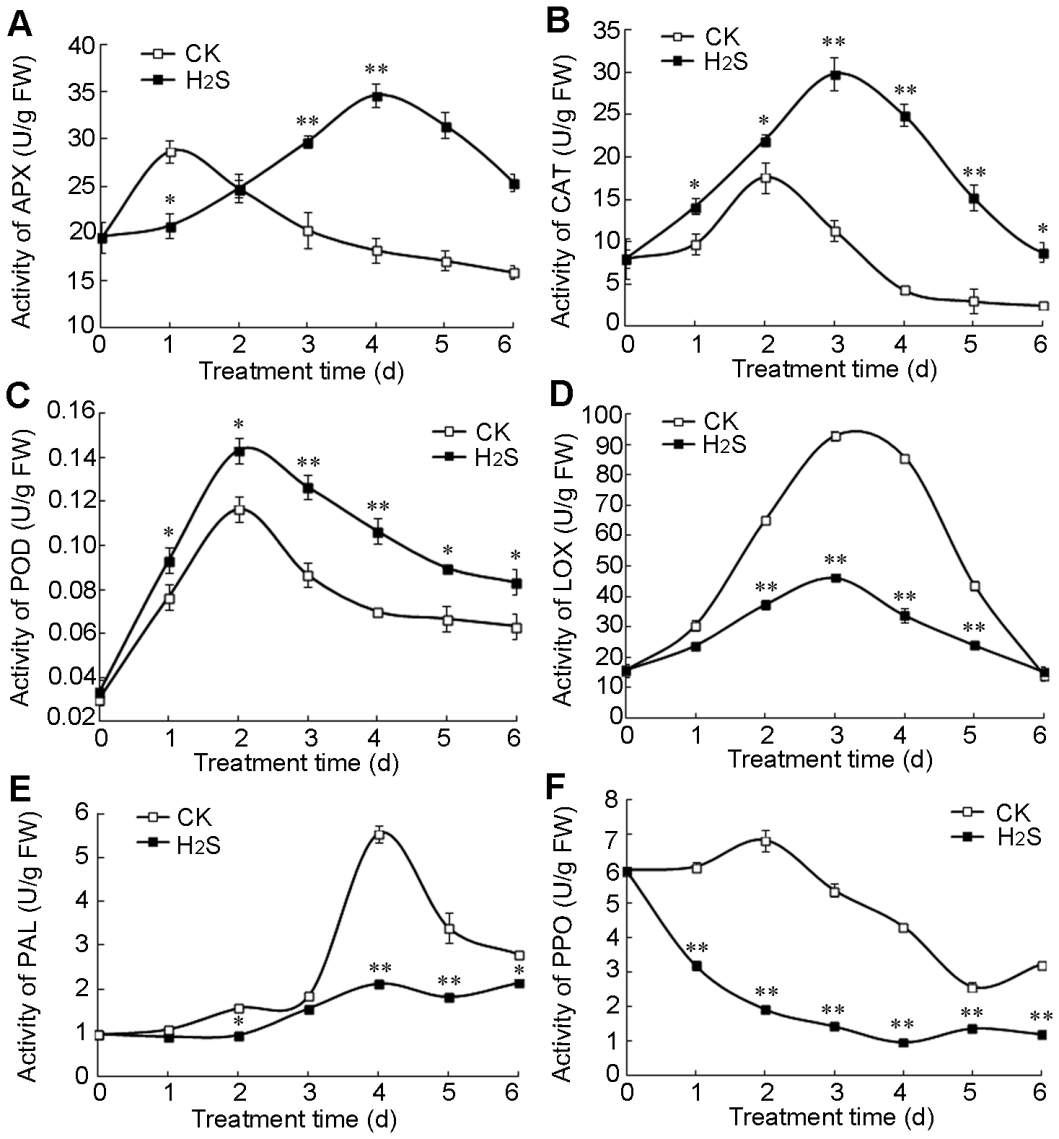
Role of H_2_S in the regulation of enzyme activities. The activities of ascorbate peroxidase (APX) (A), catalase (CAT) (B), guaiacol peroxidase (POD) (C), lipoxygenase (LOX) (D), phenylalanine ammonia lyase (PAL) (E) and polyphenol oxidase (PPO) (F) were determined in pears. Pear slices were fumigated with 2.0 mM H_2_S donor NaHS aqueous solution (H_2_S) or with water as the control groups (CK) for 0−6 d. Data are presented as means ± SD (n = 3). FW: fresh weight.

To further study the antioxidant role of H_2_S, the activities of enzymes involved in oxidative metabolism in plants, such as ascorbate peroxidase (APX), catalase (CAT), guaiacol peroxidase (POD), lipoxygenase (LOX), polyphenol oxidase (PPO) and phenylalanine ammonia lyase (PAL), are determined in pear slices. In water controls, APX activity increased rapidly and peaked on day 1 of storage and then declined to a level below that of fresh pears (Figure 4A). On the other hand, APX activity in H_2_S treatment increased steadily during the first 4 days of storage, and then decreased gradually (Figure 4A). After 3 days of storage, H_2_S treatment maintained significantly higher level of APX than water controls.

H_2_S treatment sustained significantly higher level of CAT compared with water controls (Figure 4B). Similar change of CAT was observed in water controls and H_2_S treatment. CAT activity in water controls increased and peaked on d 2, and then decreased dramatically till day 6. In contrast, in H_2_S-treated tissue it increased steadily and peaked at 3 d, followed by a decline.

Similar change pattern of POD activity were observed in water controls and H_2_S treatment (Figure 4C). POD activity increased rapidly during the first 2 d of storage regardless of treatment and then decreased gradually in both treatments (Figure 4C). However, exposure to H_2_S maintained significantly higher level of POD activity in pear slices than water treatment.

LOX, which catalyzes the hydroperoxidation of polyunsaturated fatty acids and leads to the production of MDA, was detected in pear slices (Figure 4D). LOX activity increased rapidly in water-treated slices reaching a maximum of ∼90 U/g FW after 3 d of storage followed by a steep decline to levels relative to that of fresh tissues (Figure 4D). In contrast, the rise of LOX activity in H_2_S treatment was less pronounced and peaked to about 45 U/g FW on 3 d of storage followed by a decline. In general, the H_2_S treatment maintained significantly lower level of LOX activity compared with water controls during the whole storage.

Phenylalanine ammonia lyase (PAL) and polyphenol oxidase (PPO), which participate in the synthesis of free phenolics and catalyze oxidation of phenolics into brown-colored pigments [27], [28], were also examined in pear slices. Changes in PAL activity, an enzyme that catalyzes the first step in the phenylpropanoid pathway, are shown in Figure 4E. PAL activity increased gradually during the first 3 days of storage in both treatments. Thereafter, a burst PAL activity was observed on d 4 in water controls, whereas the activity fluctuated at a lower level in H_2_S treatment. Then PAL activity in water controls decreased but was always significantly higher than that of H_2_S treatment. Changes in the activities of PPO are illustrated in Figure 4F and show that H_2_S fumigation maintained significantly lower PPO activities compared with controls during the entire storage. PPO activities increased gradually in control pear during the first 2 days of storage, and then its activity decreased steadily until d 5 with an increase on d 6. In contrast, PPO activities in H_2_S treated pear slices decreased dramatically up to d 4, and then remained stable.

### Fungicidal effects of H2S

**Figure 5:**
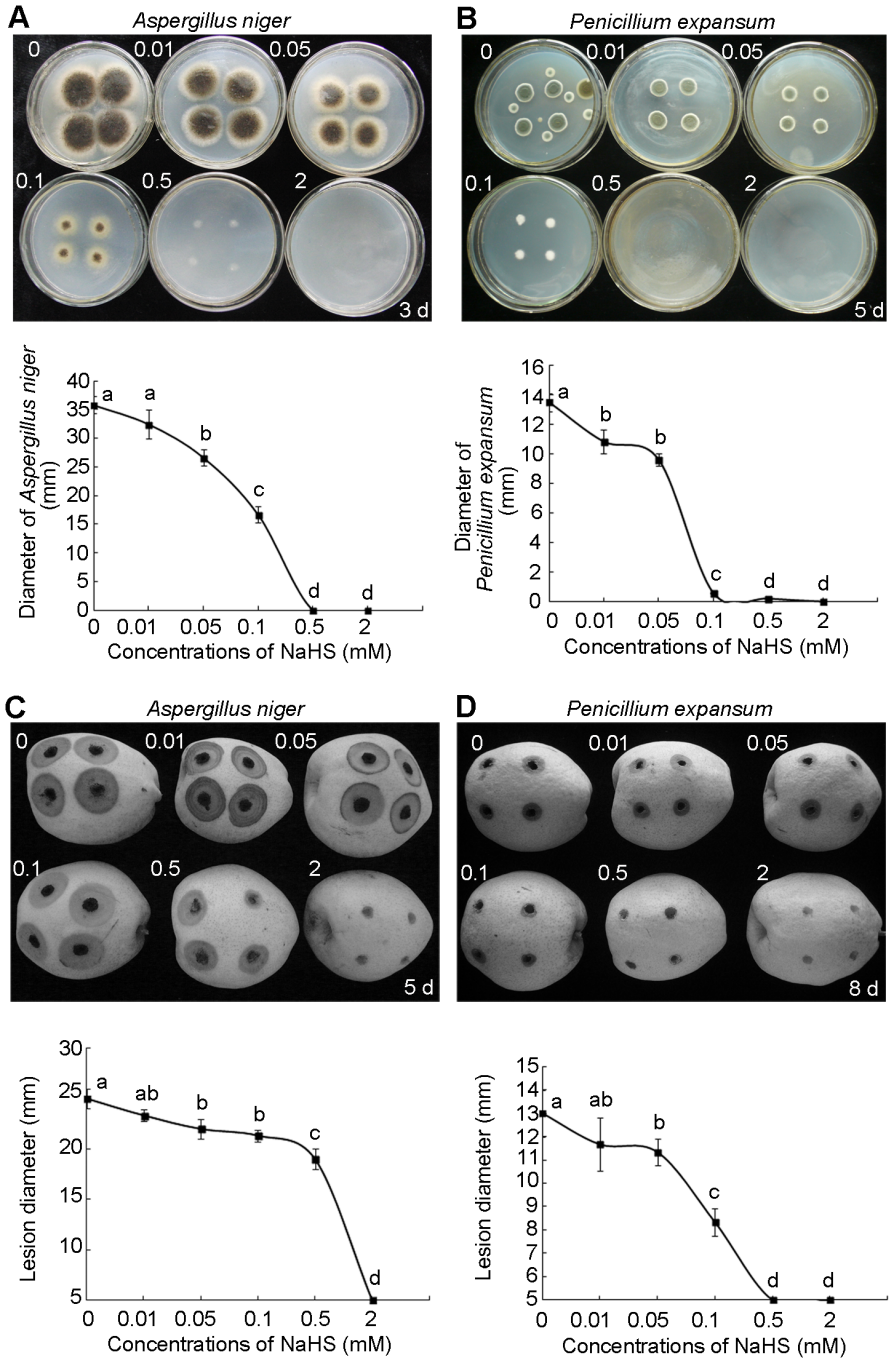
H_2_S inhibits the growth of *Aspergillus niger* and *Penicillium expansum*. *A. niger* (A) and *P. expansum* (B), isolated from pear slices, were cultured on medium and subjected to the fumigation of different concentrations of NaHS solutions for 3 and 5 days respectively. The upper photographs of (A) and (B) indicate the growth of fungi subjected to different concentrations from left to right, upper to lower 0, 0.01, 0.05, 0.1, 0.5 and 2 mM NaHS, and the lower part of figure shows the diameters of fungi clones. The upper photographs of (C) and (D) show the pears infected by *A. niger* and *P. expansum* for 5 days and 8 days, respectively. The pears were subjected to different concentrations from left to right, upper to lower 0, 0.01, 0.05, 0.1, 0.5 and 2 mM NaHS, and the lower part of (C) and (D) shows the diameters of wounds caused by fungi. Data are presented as means ± SD (n = 4). Different letters indicate significant differences (*p*<0.05) between the treatments.

To understand the phenomenon of reduced fungal growth under H_2_S fumigation as shown in Figure 1A, we isolated two pathogens *A. niger* and *P. expansum* from pears and investigated the effect of H_2_S on fungi growth in vitro and in vivo on pear fruits. Figure 5A and B show mycelium growth of *A. niger* and *P. Expansum* on medium, respectively. With increased concentrations of NaHS, the mycelium diameter of both pathogens grown on potato dextrose agar decreased and mycelium growth was severely inhibited even at 0.1 mM NaHS treatment. At 2 mM NaHS, mycelium growth of both fungi was completely inhibited suggesting a strong fungicidal effect of H_2_S on the two pathogens. The effects of H_2_S fumigation on fungal infection of pears are shown in Figure 5C and D. Higher concentrations of NaHS treatment severely inhibited the fungal propagation and reduced the lesion diameters on pears (lower part of Figure 5C and D). At 2 mM NaHS treatment, fungal infection of pears by *A. niger* and *P. expansum* was completely inhibited, suggesting an effective fungicidal role of H_2_S.

## Discussion

Fruits are perishable during storage at room temperature due to rapid ripening [29]. An important goal for pear producers is to delay fruit spoilage and enhance storability to deliver benefits to consumers [30]. In this paper, we demonstrate that the application of H_2_S via the donor NaHS prolongs the storage of pears at 20°C, which is a temperature to mimic the lack of cold storage in developing countries, implying that H_2_S acts as a regulator in the senescence of postharvest fruit.

H_2_S is produced in small amounts by some cells of the mammalian body and has a number of biological signaling functions [2]. Since H_2_S occurs naturally in the body, the environment and the gut, enzymes existed in the body are capable of detoxifying it by oxidation to harmless sulfate [2]. Besides, several enzymes are responsible for the production of H_2_S in plants, such as L-cysteine desulfhydrase, sulfite reductase and β-cyanoalanine synthase [8]. Hence, low levels of H_2_S could be tolerated to human beings [2]. In this paper, the application concentration of NaHS solution is 0.5–2.5 mM, which could release about 0.05–0.5 ppm H_2_S gas in a sealed container (data not shown). In our previous work, the endogenous H_2_S level in H_2_S-treated strawberry was about 10%–20% higher than that of water control. Thus we propose that fumigation with trace H_2_S gas released from NaHS solution on pear slices could be safe.

Our data show that overproduction of reactive oxygen species (ROS) such as H_2_O_2_ and •O_2_^−^ contributed to accelerated fresh-cut fruit senescence, an observation that is consistent with other studies showing that ROS increased during senescence [31]. Therefore, maintaining high levels of antioxidants in pear slices is important in delaying fresh-cut fruit spoilage. Fruit tissues have evolved a complex antioxidant system to alleviate and repair oxidative damage [32]. Edible coatings with antibrowning agents can effectively improve the accumulationg of antioxidants vitamin C and total phenols, and finnally alleviate the senescence of fresh-cut pears during storage [32]. Overproduction of ROS up-regulates various antioxidant enzymes such as POD, CAT and APX to degrade ROS and prevent damages induced directly or indirectly by ROS [33]. Then we found that H_2_S treatment in pear slices are able to increase the activities of APX, CAT and POD (Figure 4A, B and C), thereby alleviating the accumulation of H_2_O_2_ and •O_2_^−^ (Figure 3A and B). The H_2_S-induced increase in antioxidant enzyme activities suggests that H_2_S could maintain the antioxidant potential to ameliorate ROS production during fruit storage. Fumigation of plants with H_2_S increased the content of cysteine and glutathione as a way to detoxify atmospheric H_2_S [34]. Consistently, we observed that H_2_S treatment increased the content of cysteine in pear slice (Table 1). Glutathione, which a major cellular thiol, plays important roles in ROS metabolism [35]. Whether H_2_S also acts through the increase of glutathione to alleviate senescence of postharvest fruits, still needs further research.

The senescence of fresh-cut pear is a complex and highly regulated process that involves lipid peroxidation that can be quantitated by measuring the content of MDA, which is a good indicator of the structural integrity of plant membranes [36]. Lipid peroxidation in plant tissues is regulated in part by lipoxygenase (LOX) and increased LOX activity is found to be associated with enhanced lipid peroxidation in fruit tissue [37]. LOX can catalyze the oxygenation of polyunsaturated fatty acids into lipidhydroperoxides (LOOHs) and result in the formation of hydroperoxides [38]. In our work, H_2_S treatment reduced the activities of LOX and a concomitant lowering of MDA content was observed. We speculate that the lowered MDA content and LOX activity in H_2_S treatment maintains the membrane integrity, and thus a delay in fruit tissue senescence.

Surface browning is frequently observed in fresh-cut fruits. Phenylalanine ammonia lyase (PAL) and polyphenol oxidase (PPO) are involved in the synthesis of free phenolics and catalyse the oxidation of phenolics into brown-colored pigments, and finally contribute to surface browning of fresh-cut fruits [27], [28]. The decreased PAL and PPO activities in H_2_S fumigated pear slices might alleviate the browning of fresh-cut pears.

In plants, stress conditions generally result in enhanced proteolytic activity to mobilize nutrients out of dying tissues into actively growing tissues in plants [39]. Compared with water controls, H_2_S treatment of pear slices decreased the accumulation of free amino acids, suggesting a protective role of H_2_S in delaying proteolysis during storage.

In plants, H_2_S is released by the activity of L-cysteine desulfhydrase during pathogen attack [26], suggesting that the evolution of H_2_S could be an important strategy of the plant to combat with fungal attack. In the present study, we isolated two pathogens *A. niger* and *P. expansum* from pears and found that 0.5 mM NaHS severely inhibited the growth of two pathogens on medium or on pears. Sulfur compounds have being used as fungicide from the time of antiquity. Marsh reported that H_2_S was toxic to germinating spores [40]. Then Haneklaus et al. calculated that a minimum uptake of 10 µM H_2_S/h by the pathogen could generate a fungicidal effect [41]. Besides inhibition of germinating spores, the fungicide mechanism of H_2_S towards postharvest fungi still needs further research.

In conclusion, the experimental data lend support to the hypothesis that H_2_S plays as an antioxidant and fungicide in pear slices, leading to a delay in senescence and decay. Our study provides an alternative strategy instead of synthetic fungicides in control of postharvest pathogens. However, the mechanisms whereby H_2_S functions in plants and fungi are largely unknown and further work is required to elucidate the possible mechanism of senescence alleviation and fungicide.

## Materials and Methods

### Materials

Pears (*Pyrus pyrifolia* cv. Dangshan), harvested from commercial orchards in Dangshan, China, were purchased from supermarket in Hefei, China. The pears are available broadly in the markets in Hefei, China. Thus no specific permissions were required for these activities. Thirty pears of uniform size were selected for experiments (weight: 300±5 g, diameter: 8.0×10.0 cm). Each pear was cut into six slices longitudinally and each slice from different pears was placed in the middle part of a sealed 3 L container. Aqueous solutions of sodium hydrosulfide (NaHS) were used as an H_2_S donor. 200 mL NaHS solutions at concentrations of 0.0, 0.5, 1.0, 1.5, 2.0, 2.5, 3 and 3.5 mM were placed in the bottom of containers to fumigate the slices for 0–6 days at 20°C, which is a temperature to mimic the lack of cold storage in developing countries. The relative humidity in the container is 90–95%. After treatment, pear slices were peeled, cored and stored at −20°C for further analysis. Each experiment was repeated three times.

### Rot index of pears

Rot index was measured by Zhu and Zhou [42] and Ayala-Zavala et al. [43] with modifications. In each treatment, six pear slices were selected for investigating the number of decay fruits. All slices were classified in four ranks by the extent of rot. 0, no rot; 1, rot surface less than 10%; 2, rot surface between 10% and 30%; 3, rot surface between 30% and 50%; 4, rot surface more than 50%. The rot index was expressed by the following equation: Rot index = [∑(*rank×quantity*)/10×6]×100%. The rot classification was recorded every two days. The experiment was repeated three times.

### Fruit firmness evaluation

Fruit firmness was measured at the equatorial part of each pear slice by a 5 mm diameter flat probe with a texture analyzer (Model TA XT plus, SMS) [44]. The penetration depth was 5 mm and the cross-head speed was 5 mm/s. Fruit firmness values were an average of 6 slices of pear. Data are presented as means ± SD (n = 6).

### Measurement of soluble protein and reducing sugars

Soluble protein and reducing sugar contents were measured according to Bradford [45] and Miller [46], respectively. Fruit samples (5.00±0.05 g) were ground in 5 mL of phosphate buffer (pH 7.0, 200 mM). The homogenate was centrifuged at 10,000 g for 30 min, and the supernatant was used for determination of soluble protein and reducing sugar content. For soluble protein, 0.1 mL of supernatant was mixed with 0.9 mL of dH_2_O and 5 mL of Coomassie brilliant blue. Absorbance was recorded at 595 nm after 5 min. The results are expressed as mg/g FW (fresh weight). Each experiment was repeated three times.

Reducing sugar was measured via the dinitrosalicylic acid method. The supernatant (0.2 mL) was mixed with 1.5 mL of 3,5-dinitrosalicylic acid and 1.8 mL of distilled water; the mixture was heated at 100°C for 5 min, cooled, and added to 25 mL with distilled water. Reducing sugars were determined spectrophotometrically at 540 nm and the results are expressed as mg/g FW (fresh weight). Each experiment was repeated three times.

### Measurement of free amino acids

For amino acid measurement, 5 g pear tissue was ground in 5 mL ethanol. The homogenate was centrifuged at 10,000 g for 10 min and the supernatant was heated at 80°C to removal ethanol. The residue was then diluted in water and filtered through a membrane (0.45 µm pore) and used for free amino acid assay. Free amino acids were analyzed with a Beckman Coulter S433D amino acid analyzer [47].

### Determination of •O_2_^−^, H_2_O_2_ and MDA

Contents of •O_2_^−^, H_2_O_2_ and MDA were determined according to the methods described by Hu et al [11].

### Activity assays of LOX, CAT, APX, POD, PAL and PPO

Lipoxygenase (LOX, EC 1.13.11.12) activity was determined following the description by Surrey [48], and catalase (CAT, EC 1.11.1.6) and ascorbate peroxidase (APX, EC 1.11.1.11) were assayed according to Garcia-Limones et al [49]. Pear tissues (5 g) were homogenized in 2.5 mL ice-cold 0.15 M phosphate buffer (pH 7.6) containing 1.0 mM ethylenediaminetetraacetic acid and 1% polyvinyl pyrrolidone. The homogenate was centrifuged at 10,000 g at 4°C for 30 min. The supernatant was used for activity measurement. For CAT activities, H_2_O_2_ was used as substrate. CAT activities were assayed by monitoring the decrease in absorbance at 240 nm. APX was determined in the presence 2.0 mM ascorbic acid and 2.0 mM ethylenediaminetetraacetic acid by measuring the decrease in absorbance at 290 nm. Analysis of POD was based on the oxidation of guaiacol using hydrogen peroxide by monitoring an increase in absorbance at 470 nm [11]. The reaction mixture contained 5.0 mL of 50 mM potassium phosphate buffer (pH 6.0), 1 mM of 1% hydrogen peroxide, 1 mM of 1% guaiacol and 10–20 µL of enzyme extract. One unit of CAT, APX or POD was defined as an increase or decrease of 0.01 in absorbance per minute under the assay conditions. The activities of antioxidant enzymes were expressed as U/g FW (fresh weight). Each experiment was repeated three times.

Activity of phenylalanine ammonia lyase (PAL) was determined by procedures described by Beaudoin-Eagan and Thorpe [50]. One unit of PAL activity was defined as a change of 0.01 OD value in absorbance at 290 nm per minute. The results were expressed as U/g FW (fresh weight). Each experiment was repeated three times.

Activity of polyphenol oxidase (PPO) was determined by procedures described by Benjamin and Montgomery [51]. Pear samples (2.00±0.05 g) were homogenized with 5.0 mL of sodium phosphate buffer (50 mM, pH 6.8). After centrifugation, the supernatant was used for the activity assay using catechol as substrate. One unit of PPO activity was defined as an increase of 0.01 OD value in absorbance at 410 nm per minute. The results were expressed as U/g FW (fresh weight). Each experiment was repeated three times.

### Effect of H_2_S on fungal growth

The fungi *Aspergillus niger* and *Penicillium expansum* were isolated from the surface of pear fruits, and cultured on a potato-dextrose agar (PDA) medium (containing the extract from 200 g of boiled potato, 20 g of glucose and 20 g of agar in 1 L of distilled water) at 28°C in the dark [52]. Spore suspensions were prepared by flooding 6-day-old sporulating cultures of *A. niger* and *P. expansum* with sterile distilled water. The spore concentrations of the pathogen were determined with a hemacytometer and diluted with sterile distilled water to 1×10^5^ spores/mL. Four aliquots of spore suspension (5 µL) were placed on 7 mm diameter Petri dishes maintained in sealed 3 L containers. 300 mL NaHS solutions at concentrations of 0.0, 0.01, 0.05, 0.1, 0.5, 2 mM were placed in the bottom of the sealed containers at 20°C with a relative humanity of 90−95% to fumigate fungi for the number of days indicated in the figure legends. For fungal infection of pears, pear fruits were wounded, four wounds per pear (5 mm diameter and 5 mm deep), using the tip of a sterile borer [37]. 20 µL spore suspension at 1×10^5^ spores/mL of *A. niger* and *P. expansum* were inoculated onto each wound. After air-drying, the pears were stored under the same conditions as for fungi and fumigated with different concentrations of NaHS. Each experiment was repeated three times.

### Statistical analysis

Each experiment was repeated three times. Significance was tested by one-way or two-way ANOVA, and the results were expressed as the mean values ± SD of three independent experiments. Fisher's least significant differences (LSD) were calculated following a significant (P<0.01 or P<0.05) *t* test.
